# Butorphanol with oxygen insufflation improves cardiorespiratory function in field-immobilised white rhinoceros (*Ceratotherium simum*)

**DOI:** 10.4102/jsava.v86i1.1276

**Published:** 2015-08-12

**Authors:** Anna Haw, Markus Hofmeyr, Andrea Fuller, Peter Buss, Michele Miller, Gregory Fleming, Leith Meyer

**Affiliations:** 1 School of Physiology, Faculty of Health Sciences, University of the Witwatersrand, South Africa; 2 South African National Parks, Skukuza, South Africa; 3 Division of Molecular Biology and Human Genetics, DST/NRF Centre of Excellence for Biomedical Tuberculosis, Stellenbosch University, South Africa; 4 Disney's Animal Programs and Environmental Initiatives, Lake Buena Vista, United States; 5 Department of Paraclinical Sciences, University of Pretoria, Onderstepoort, South Africa

## Abstract

Opioid-induced immobilisation results in severe respiratory compromise in the white rhinoceros (*Ceratotherium simum*). The effectiveness of oxygen insufflation combined with butorphanol in alleviating respiratory depression in free-ranging chemically immobilised white rhinoceroses was investigated. In this prospective intervention study 14 free-ranging white rhinoceroses were immobilised with a combination of etorphine, azaperone and hyaluronidase. Six minutes (min) after the animals became recumbent, intravenous butorphanol was administered and oxygen insufflation was initiated. Previous boma trial results were used for comparison, using repeated measures two-way analysis of variance. The initial immobilisation-induced hypoxaemia in free-ranging rhinoceroses (arterial partial pressure of oxygen [P_a_O_2_] 35.4 mmHg ± 6.6 mmHg) was similar to that observed in boma-confined rhinoceroses (P_a_O_2_ 31 mmHg ± 6 mmHg, *n* = 8). Although the initial hypercapnia (P_a_CO_2_ 63.0 mmHg ± 7.5 mmHg) was not as severe as that in animals in the boma trial (79 mmHg ± 7 mmHg), the field-immobilised rhinoceroses were more acidaemic (pH 7.10 ± 0.14) at the beginning of the immobilisation compared with boma-immobilised rhinoceroses (pH 7.28 ± 0.04). Compared with pre-intervention values, butorphanol with oxygen insufflation improved the P_a_O_2_ (81.2 mmHg ± 23.7 mmHg, *p* < 0.001, 5 min vs 20 min), arterial partial pressure of carbon dioxide (55.3 mmHg ± 5.2 mmHg, *p* < 0.01, 5 min vs 20 min), pH (7.17 ± 0.11, *p* < 0.001, 5 min vs 20 min), heart rate (78 breaths/min± 20 breaths/min, *p* < 0.001, 5 min vs 20 min) and mean arterial blood pressure (105 mmHg ± 14 mmHg, *p* < 0.01, 5 min vs 20 min). Oxygen insufflation combined with a single intravenous dose of butorphanol improved oxygenation and reduced hypercapnia and acidaemia in immobilised free-ranging white rhinoceroses.

## Introduction

A key component of the conservation of white rhinoceroses (*Ceratotherium simum*) relies on the ability to safely immobilise these large pachyderms for translocation, dehorning, identity marking and veterinary treatments. Potent mu-agonist opioids, such as etorphine hydrochloride, are the only category of drugs capable of inducing rapid and reversible immobilisation of free-ranging white rhinoceroses. However, a drawback of use of these potent opioids is respiratory depression characterised by hypoventilation, hypoxaemia, hypercapnia and acidaemia (Burroughs, Meltzer & Morkel 2012; Bush *et al.* 2004; Hattingh & Knox 1994; Heard, Olsen & Stover 1992; Portas 2004; Wenger *et al.* 2007).

The massive size of the white rhinoceros, and in particular its large digestive tract together with muscle rigidity are thought to be significant factors in reducing the ability of the animal to breathe adequately during recumbency (Cornick-Seahorn *et al.* 1995). As a result, potentially life-threatening hypoxaemia is one of the most significant side-effects in etorphine-immobilised free-ranging white rhinoceroses (Fahlman, Foggin & Nyman 2004; Heard *et al.* 1992; Kock *et al.* 1995; Raath 1999).

In a series of controlled experiments in captive white rhinoceroses housed in bomas (small enclosures to hold wild animals temporarily) it was demonstrated that intravenous butorphanol administered at the beginning of the immobilisation period, together with oxygen insufflation supplied throughout immobilisation, provided the best cardiorespiratory-supportive treatment for immobilised, laterally recumbent rhinoceroses (Haw *et al.* 2014). However, outside the experimental setting most rhinoceroses are wild caught in the field. The physiology of field-immobilised rhinoceroses may differ substantially from that of boma-kept animals, particularly if exercise and additional stress are imposed by a vehicle or helicopter chase to which an animal is not accustomed. It is therefore not known whether a cardiorespiratory treatment found to be effective in immobilised, boma-kept rhinoceroses would be as effective in free-ranging rhinoceros.

The aim of the present study was to establish whether butorphanol combined with oxygen insufflation would afford the same positive results in field-immobilised rhinoceroses as it did in boma-kept white rhinoceroses.

## Research method and design

### Study area and sample population

The study animals were 14 sub-adult (3–7 year-old) male white rhinoceroses, which were immobilised in Kruger National Park (-24.98984 S, 31.59263 E, altitude 317 m) during February 2013. All immobilisations took place between 06:00 and 12:30 and ambient temperature was between 22 °C and 32 °C. Barometric pressure, measured by the on-board barometer of the blood gas analyser, ranged from 717.1 mmHg to 748.3 mmHg. All animals appeared healthy based on body condition, which was confirmed on retrospective haematology and serum biochemical analyses.

### Design

Fourteen rhinoceroses were immobilised with a combination of 2.0 mg – 3.5 mg etorphine hydrochloride (M99^®^, Novartis, Kempton Park, South Africa, 9.8 mg/mL), 30.0 mg – 52.5 mg azaperone (Stressnil^®^, Janssen Pharmaceuticals Ltd, Halfway House, South Africa, 40 mg/mL), and 2500 IU hyaluronidase (lyophilised hyalase, Kyron Laboratories, Benrose, South Africa). Each rhinoceros was located and darted from a helicopter, and drug doses were determined by an experienced veterinarian (M.H.) based on estimated body mass.

Immobilising drugs were administered using a 3 mL plastic dart with a 60 mm needle, fired by a carbon dioxide-powered dart-gun (Dan-Inject, International S.A., Skukuza, South Africa). Once the animal was safe to handle a ground crew approached, placed a blindfold on the rhinoceros, and positioned the animal in lateral recumbency (time 0), elevating the head with padding to ensure that the lower nostril remained patent for unobstructed ventilation. One observer in the helicopter estimated the distance that the rhinoceros ran before and after darting by counting 100 m segments on the ground with the aid of the helicopter's global positioning system. Signs of induction from darting to recumbency, including slowing and ataxia, were observed from the helicopter and times recorded.

If an animal was not recumbent within 15 min of darting it was excluded from the study and the effects of the immobilising agent immediately reversed. The speed at which the rhinoceros ran after dart placement was calculated as the estimated distance (metres) travelled divided by the time (seconds) it took for the animal to come to a complete halt.

Each rhinoceros received intravenous butorphanol (15 mg/mg etorphine) 6 min after it was positioned in lateral recumbency, combined with tracheal oxygen insufflation (30 L/min, see details on administration below); oxygen insufflation started at 6 min and continued throughout the recumbent period. Clinical data and samples were collected at 5 min after animals became laterally recumbent (1 minute before the intervention) and every 5 min thereafter for a 25-minute immobilisation period.

### Clinical monitoring and data collection

Heart rate was measured directly by auscultation and counting the number of beats over one minute. Body temperature was measured with a thermocouple probe inserted 10 cm into the rectum and connected to a thermometer (BAT-12, Physitemp Instruments Clifton, New Jersey, United States of America [USA]). Respiratory rate was measured by observing and counting thoracic and abdominal excursions and feeling for expired air at the nares, over one minute.

The level of immobilisation was assessed by a veterinarian (M.H.) observing whole-body and ear movements, which were scored at 5, 10, 15, 20 and 25 min. The immobilisation score ranged from 1 (no immobilising or sedative effect) to 6 (excessive immobilisation depth with respiration < 3 breaths/min). Level 3 indicated a safe standing sedation, whilst levels 4 and 5 indicated recumbent immobilisation with or without ear movement, respectively.

The medial auricular artery was catheterised using a catheter (22G × 1”, Nipro Safelet Cath, Nipro Corporation) secured in place with superglue (SuperGlue, Loctite^®^). A three-way stopcock valve with 10 cm compliant tube extension (Mediflexo, Eastern Medikit Ltd) was attached to the catheter to allow continuous direct blood pressure measurements via a pressure transducer (Deltran II, Utah Medical Products Inc., USA) attached to a custom-made portable processor (IntraTorr, Edenvale, South Africa) and easy access for arterial samples. A 0.5 mL sample was collected anaerobically into 1 mL pre-heparinised syringes at 5, 10, 15, 20 and 25 min after the rhinoceros became laterally recumbent.

Arterial pH, partial pressure of carbon dioxide (P_a_CO_2_), partial pressure of oxygen (P_a_O_2_) and lactate were measured, whilst haemoglobin oxygen saturation (S_a_O_2_) and bicarbonate (HCO_3_) were calculated immediately, using a portable precalibrated blood gas analyser with self-calibrated test cards (EPOC^® ^Portable analyser system and EPOC^®^ BGEM test cards, Kyron Laboratories, Johannesburg, South Africa). The blood gas variables were reported at 37 °C. The catheter was maintained with a heparinised (heparin 5000 IU/mL; Fresenius, Port Elizabeth, South Africa) saline flush and 1 mL – 2 mL of blood was discarded before collection of each sample.

Oxygen (100% medical oxygen, African Oxygen Limited [Afrox], Johannesburg, South Africa) was delivered at30 L/min using an equine stomach tube (9.5 mm outer diameter × 213 cm, Kyron Laboratories) inserted nasotracheally as described previously (Bush *et al.* 2004). The tube was inserted to a predetermined length, which ensured delivery of oxygen into the distal end of the trachea; air movement was felt to verify that the tube was in the trachea and not the oesophagus. Custom-designed tubing (Hospital Servicing Consultants cc, Lonehill, South Africa) was used to connect the stomach tube to two 15 L/min oxygen flow metres operating in parallel.

Silver duct tape was applied to the rhinoceros’ horn and back leg to mark the animal temporarily and ensure that it was not re-immobilised during the study.

Twenty-six min into the recumbent period the rhinoceros was stimulated to stand and guided into a crate for weighing. Quality of arousal was assessed by M.H. according to the amount of stimulation needed to get the animal into a standing position and the ease with which the animals could be guided into a crate. The score was based on an incremental scale from 1 (animal gets up quickly, too awake and cannot be safely handled) to 5 (animal struggles to get up even after excessive stimulation, is unable to walk and becomes recumbent). A score of 3 (animal gets up after moderate stimulation and when standing can be safely handled and walked with stimulation) was considered ideal.

About 40 min after initial recumbency the effects of etorphine were reversed using the full-opioid antagonist naltrexone (Naltrexone, 50 mg/mL, Kyron Laboratories, Johannesburg), administered intravenously into an auricular vein at 20 mg/mg etorphine. The rhinoceros was then released from the weighing crate.

### Comparison to boma-held rhinoceroses

Previously reported findings from a study on eight individually housed sub-adult male white rhinoceroses that each received four different treatment interventions at two-week intervals were used for comparison (Haw *et al.* 2014). Each rhinoceros was immobilised with the same drug combinations and dose rates as used in this field study. However, the boma-kept rhinoceroses were unobtrusively darted from a platform above each boma, rather than from a helicopter. Similar to this field study, treatment interventions in the boma trial were administered 6 min after the rhinoceros became laterally recumbent and comprised: (1) intravenous butorphanol (15 mg/mg etorphine), (2) tracheal oxygen insufflation at 30 L/min, which started at 6 min and continued throughout the immobilisation period, (3) intravenous butorphanol (15 mg/mg etorphine) combined with tracheal oxygen insufflation (butorphanol + oxygen) and (4) intravenous sterile water (control).

**FIGURE 1:**
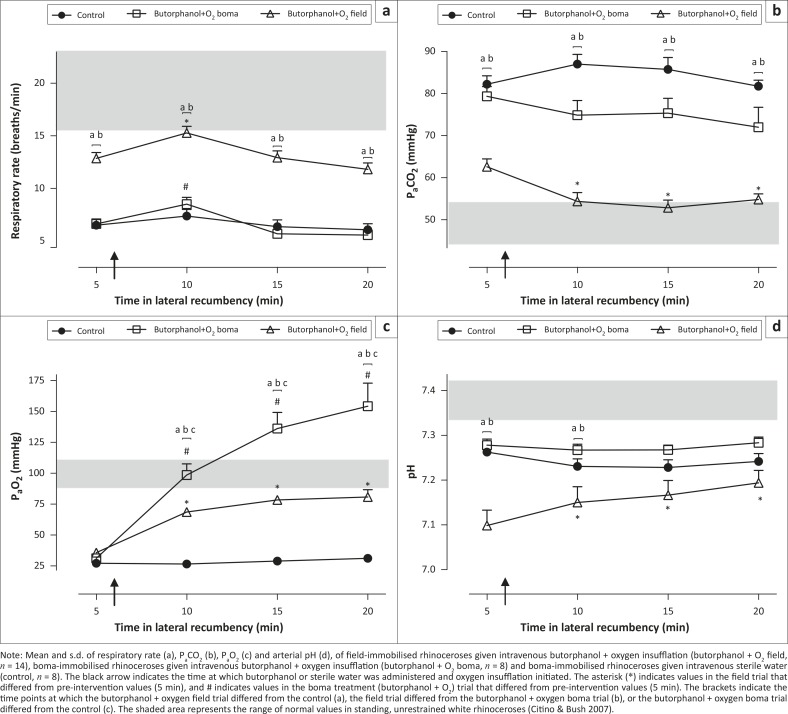
(a) Respiratory rate, (b) P_a_CO_2_, (c) P_a_O_2_ and (d) pH. Note: Mean and s.d. of respiratory rate (a), P_a_CO_2_ (b), P_a_O_2_ (c) and arterial pH (d), of field-immobilised rhinoceroses given intravenous butorphanol + oxygen insufflation (butorphanol + O_2_ field, *n* = 14), boma-immobilised rhinoceroses given intravenous butorphanol + oxygen insufflation (butorphanol + O_2_ boma, *n* = 8) and boma-immobilised rhinoceroses given intravenous sterile water (control, *n* = 8). The black arrow indicates the time at which butorphanol or sterile water was administered and oxygen insufflation initiated. The asterisk (*) indicates values in the field trial that differed from pre-intervention values (5 min), and # indicates values in the boma treatment (butorphanol + O_2_) trial that differed from pre-intervention values (5 min). The brackets indicate the time points at which the butorphanol + oxygen field trial differed from the control (a), the field trial differed from the butorphanol + oxygen boma trial (b), or the butorphanol + oxygen boma trial differed from the control (c). The shaded area represents the range of normal values in standing, unrestrained white rhinoceroses (Citino & Bush 2007).

**FIGURE 2:**
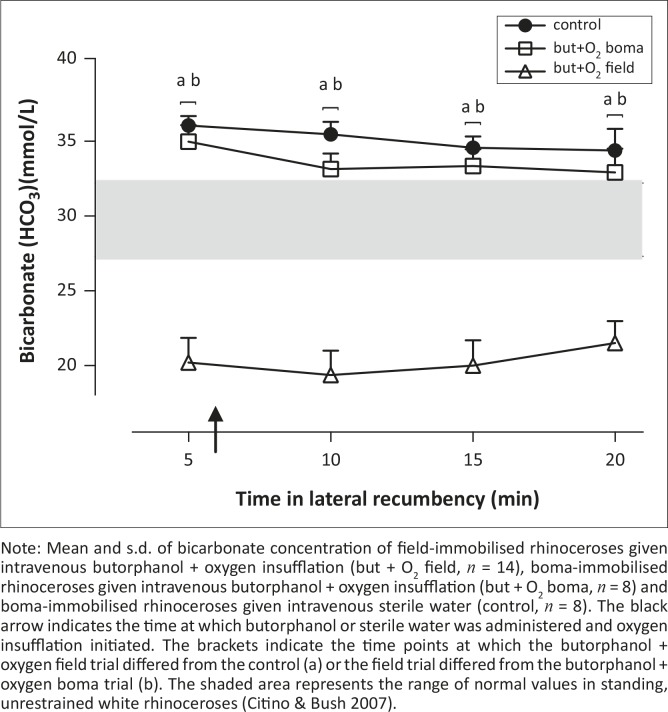
Bicarbonate concentration (HCO_3_) in immobilised white rhinoceroses. Note: Mean and s.d. of bicarbonate concentration of field-immobilised rhinoceroses given intravenous butorphanol + oxygen insufflation (but + O_2_ field, *n* = 14), boma-immobilised rhinoceroses given intravenous butorphanol + oxygen insufflation (but + O_2_ boma, *n* = 8) and boma-immobilised rhinoceroses given intravenous sterile water (control, *n* = 8). The black arrow indicates the time at which butorphanol or sterile water was administered and oxygen insufflation initiated. The brackets indicate the time points at which the butorphanol + oxygen field trial differed from the control (a) or the field trial differed from the butorphanol + oxygen boma trial (b). The shaded area represents the range of normal values in standing, unrestrained white rhinoceroses (Citino & Bush 2007).

Arterial samples for blood gas analyses and non-invasive cardiorespiratory measurements (respiratory rate and heart rate) were taken before the intervention, at 5 min, and every 5 min thereafter for 20 min during the immobilisation period, using the same methods described above. The blood gas analyser used in the boma study (Roche OPTI CCA Analyzer and OPTI cassette B, Kat Medical, Johannesburg) was different from that used in this field study, which recorded additional variables such as plasma lactate concentration. In order to test agreement between the two analysers, simultaneous measurements from 35 samples obtained from immobilised rhinoceroses were compared. P_a_O_2_, P_a_CO_2_, pH and HCO_3_ values from each analyser were significantly and strongly correlated (*p* < 0.0001; *r*^2^ = 0.97, 0.74, 0.97 and 0.94 for P_a_O_2_, P_a_CO_2_, pH and HCO_3_ respectively). The mean bias and limits of agreements between each analyser for each variable were small relative to the statistical differences found in Figures 1 and 2; thus values obtained from each analyser were used without correction.

As butorphanol + oxygen yielded the most favourable results in the boma study, this same protocol was retested in the field study. For logistical and ethical reasons (the boma trials showed critically abnormal physiological parameters in control animals) a control trial was not conducted in the field. Therefore results recorded from this field trial were compared with the boma treatment (butorphanol + oxygen) group and the boma control (sterile water) group.

### Data analysis

Statistical analyses were performed using GraphPad Prism version 4.00 for Windows (GraphPad Software, San Diego, California, USA). All results are expressed as mean ± standard deviation (s.d.) and *p* < 0.05 was considered significant. For P_a_O_2_, P_a_CO_2_, pH, respiratory rate and heart rate an unpaired repeated measures two-way analysis of variance (ANOVA) followed by Bonferroni post-tests was used to test for differences between responses to butorphanol + oxygen in field-immobilised rhinoceros, butorphanol + oxygen in boma-immobilised rhinoceros, and sterile water (control) in boma-immobilised rhinoceros at 5, 10, 15 and 20 min and between time points for each intervention. Although field-immobilised rhinoceroses were recumbent for 25 min, data are represented for the first 20 min of lateral recumbency, to allow for direct comparison to the boma study in which rhinoceroses were recumbent for only 20 min.

Pearson's correlation was performed to test for a relationship between plasma lactate at 5 min and the average estimated distance travelled by and average calculated running speed of each rhinoceros after the dart was fired. A repeated measures one-way ANOVA followed by Tukey's multiple comparison was used to test for differences in lactate concentrations at 5, 10, 15 and 20 min in the field-immobilised rhinoceroses.

## Results

Rectal temperatures for field-immobilised rhinoceros ranged from 37.2 °C to 39.2 °C. The average body mass of the 14 field-captured rhinoceroses was 1272 kg ± 227 kg and each rhinoceros was estimated to be in the correct body mass bracket according to the body mass to dose table (Haw *et al.* 2014). No mortality occurred in the rhinoceroses that were studied.

### Induction period

Free-ranging rhinoceroses ran an estimated average distance of 1400 m ± 800 m before darting. The helicopter was used to herd rhinoceroses before dart placement, to ensure that once they were darted a ground crew could easily access the immobilised animals. Once darted the rhinoceros ran a further 1300 m ± 300 m before halting. The total combined distance ranged from 1000 m to 4400 m. The estimated speed the rhinoceros ran from darting to halting ranged from 2.5 m/s to 8.3 m/s (average 4.9 m/s ± 1.9 m/s). The administration of etorphine, azaperone and hyaluronidase led to rapid immobilisation of free-ranging rhinoceros, with recumbency attained in 7 min ± 2 min after darting. The time to recumbency was similar in the boma-immobilised rhinoceroses in both the control (5 min ± 1 min) and the butorphanol + oxygen trials (6 min ± 1 min). Throughout the recumbent period all field-immobilised rhinoceroses had an immobilisation level of 4 (recumbent with occasional ear movements). Both the treatment and control boma-immobilised rhinoceroses also had a median immobilisation score of 4.

### Respiratory rate

Immobilisation caused hypopnoea (13 breaths/min ± 2 breaths/min, normal range 16–23 breaths/min [Citino & Bush 2007], Figure 1a) in field-immobilised rhinoceroses. After the administration of butorphanol and oxygen (at 6 min) there was an initial improvement in respiratory rate (15 breaths/min ± 2 breaths/min, *p* < 0.01, 5 min vs. 10 min, Figure 1a), but this improvement was short-lived with the respiratory rate decreasing again to 13 breaths/min ± 2 breaths/min at 15 min. A similar pattern in respiratory rate was observed in boma-immobilised rhinoceroses given the same supportive treatment (Figure 1a). However, the hypopnoea in the boma-immobilised rhinoceroses (treatment group; 7 breaths/min ± 2 breaths/min at 5 min, *p* < 0.001) was more severe than in the field. The respiratory rate of boma-immobilised rhinoceroses given butorphanol + oxygen was not different from that of boma-immobilised rhinoceroses that did not receive any supportive treatment (control). Overall, immobilised rhinoceroses in the field (given butorphanol + oxygen) had respiratory rates about twice those of boma-immobilised rhinoceros in both the treatment (butorphanol + oxygen) and control groups, throughout the immobilisation period [*F*_(2,27)_ = 62.41, *p* < 0.001].

### Partial pressure of arterial carbon dioxide (P_a_CO_2_)

Field-immobilised rhinoceroses were hypercapnic at 5 min (63.0 mmHg ± 7.5 mmHg, normal range 44.4 mmHg –53.7 mmHg [Citino & Bush 2007], Figure 1b). Administration of butorphanol with oxygen insufflation led to an immediate decrease in P_a_CO_2_ (54.7 mmHg ± 8.5 mmHg at 10 min, *p* < 0.01 vs. 5 min, Figure 1b). This lower arterial carbon dioxide concentration was sustained throughout the immobilisation period (P_a_CO_2_ at 20 min 55.3 mmHg ± 5.2 mmHg, *p* < 0.01, 5 min vs. 20 min). P_a_CO_2_ values in the field-immobilised rhinoceroses were also lower [*F*_(2,28)_ = 60, *p* < 0.001] than those of the boma control and treatment (butorphanol + oxygen) trials at all time points, including the period before the treatment intervention was administered. In boma-immobilised rhinoceroses butorphanol + oxygen did not lower the P_a_CO_2_ concentration compared with pre-intervention values [*F*_(3,21)_ = 1.3, *p* = 0.3], and there was no difference in the arterial carbon dioxide concentrations between boma-kept animals in the control and treatment (butorphanol + oxygen) groups.

### Partial pressure of arterial oxygen (P_a_O_2_)

Chemical immobilisation led to severe hypoxaemia at 5 min (P_a_O_2_ 35.4 mmHg ± 6.6 mmHg, normal range 90.2 mmHg –108.6 mmHg [Citino & Bush 2007], Figure 1c) in field-immobilised white rhinoceroses. A similar degree of hypoxaemia was observed in boma-immobilised rhinoceroses (P_a_O_2_ 31 mmHg ± 6 mmHg, *n* = 8, Figure 1c). Butorphanol combined with oxygen insufflation had an immediate positive effect on oxygenation in field-immobilised rhinoceroses (P_a_O_2_ at 10 min 68.9 mmHg ± 7.2 mmHg, *p* < 0.01, 5 vs. 10 min, Figure 1c) and improved arterial oxygenation compared with control boma-immobilised rhinoceroses at each time point following the intervention (*p* < 0.05 at 10 min; *p* < 0.01 at 15 min and 20 min). Similarly, butorphanol + oxygen had a profound positive effect in boma-immobilised rhinoceroses (P_a_O_2_ at 10 min 99 mmHg ± 26 mmHg, *n* = 8, Figure 1c), completely correcting hypoxaemia, with P_a_O_2_ values reaching 154 mmHg ± 53 mmHg 20 min into the immobilisation period. However, in the field-immobilised rhinoceroses given the same treatment (butorphanol + oxygen), oxygenation was not completely corrected (81.2 mmHg ± 23.7 mmHg at 20 min), with only three out of 14 rhinoceros achieving arterial oxygen concentrations greater than 90 mmHg at 20 min.

### Arterial pH

Five min into the recumbent period all field-immobilised rhinoceroses were acidaemic (pH 7.10 ± 0.14, normal range 7.35–7.43 [Citino & Bush 2007], Figure 1d). Similar initial acidaemia was observed in boma-immobilised rhinoceroses (pH 7.28 ± 0.04 at 5 min), but was not as severe as in the field-immobilised rhinoceroses (*p* < 0.05, field vs. boma rhinoceroses at 5 min). In the field pH improved after administration of butorphanol + oxygen, with all pH values higher [*F*_(3,39) _= 34.57, *p* < 0.01, Figure 1d] than pre-intervention values. However, at 20 min the animals were still acidaemic (pH 7.17 ± 0.11). The pH in boma-immobilised rhinoceroses given the same supportive intervention as those in the field did not improve [*F*_(3,21)_ = 0.55, *p* = 0.66], resulting in no difference in arterial pH between the field- and boma-immobilised rhinoceroses at 20 min (*p* > 0.05). Similarly, field-immobilised rhinoceroses were initially more acidaemic than the control group in the boma (*p* < 0.01), but by the end of the immobilisation period (20 min) there was no difference in pH between the field-immobilised treatment group and the control group in the boma (*p* > 0.05).

### Bicarbonate and lactate

**FIGURE 3:**
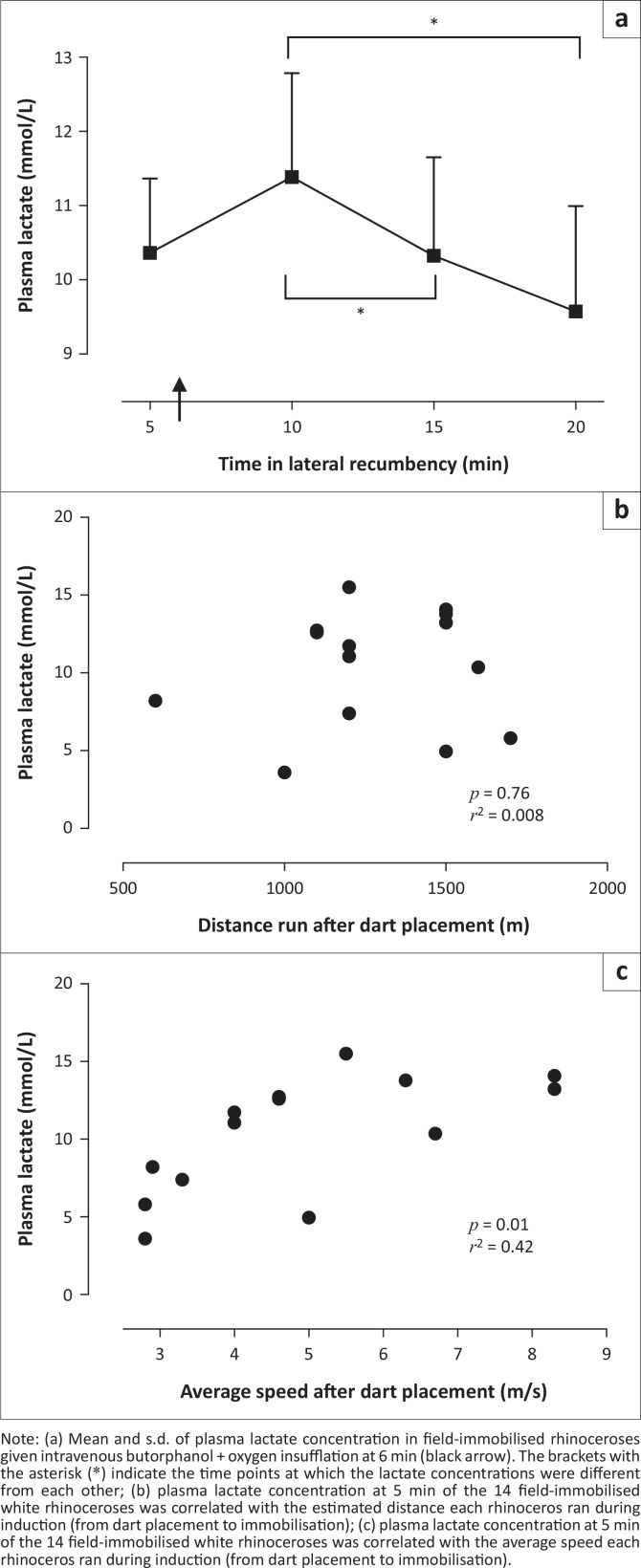
(a–c) Plasma lactate. Note: (a) Mean and s.d. of plasma lactate concentration in field-immobilised rhinoceroses given intravenous butorphanol + oxygen insufflation at 6 min (black arrow). The brackets with the asterisk (*) indicate the time points at which the lactate concentrations were different from each other; (b) plasma lactate concentration at 5 min of the 14 field-immobilised white rhinoceroses was correlated with the estimated distance each rhinoceros ran during induction (from dart placement to immobilisation); (c) plasma lactate concentration at 5 min of the 14 field-immobilised white rhinoceroses was correlated with the average speed each rhinoceros ran during induction (from dart placement to immobilisation).

Throughout the immobilisation period field-immobilised rhinoceroses had significantly lower bicarbonate concentrations than both the treatment and control boma-immobilised rhinoceroses [*F*_(2,27)_ = 36.99, *p* < 0.0001] (Figure 2). At the beginning of the immobilisation period 12 of 14 (86%) of the rhinoceroses in the field had high (above 5 mmol/L) lactate concentrations. The lactate concentrations in the rhinoceroses decreased during the immobilisation period [*F*_(2,25)_ = 5.02, *p* = 0.02], with values at 15 min (10.3 mmol/L ± 5.0 mmol/L) and 20 min (9.6 mmol/L ± 5.0 mmol/L) lower than those at 10 min (11.4 mmol/L ± 5.2 mmol/L) (*p* < 0.05, Figure 3a). Plasma lactate concentration was not correlated to the estimated distance run by the rhinoceros after dart placement (*p* = 0.76, *r*^2^ = 0.008, Figure 3b). However, the average speed at which the rhinoceros ran after dart placement was positively correlated to plasma lactate concentration at 5 min (*p* = 0.01, *r*^2^ = 0.42, Figure 3c).

### Heart rate

**FIGURE 4:**
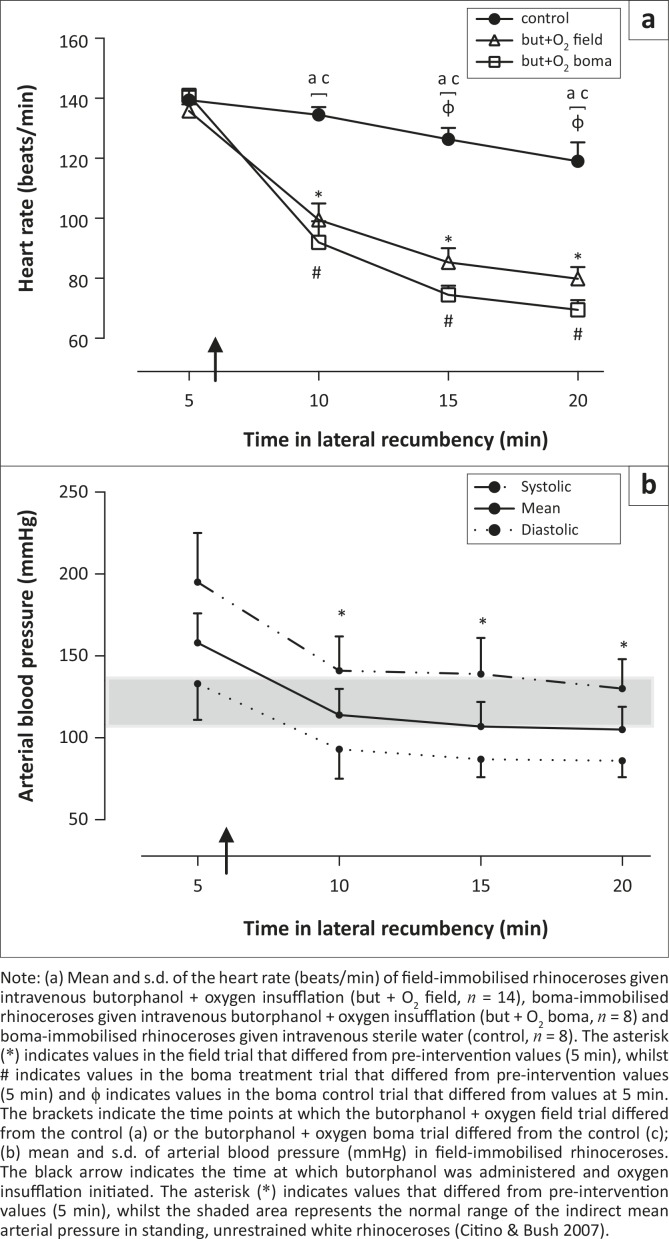
(a, b) Heart rate and arterial blood pressure. Note: (a) Mean and s.d. of the heart rate (beats/min) of field-immobilised rhinoceroses given intravenous butorphanol + oxygen insufflation (but + O_2_ field, *n* = 14), boma-immobilised rhinoceroses given intravenous butorphanol + oxygen insufflation (but + O_2_ boma, *n* = 8) and boma-immobilised rhinoceroses given intravenous sterile water (control, *n* = 8). The asterisk (*) indicates values in the field trial that differed from pre-intervention values (5 min), whilst # indicates values in the boma treatment trial that differed from pre-intervention values (5 min) and ϕ indicates values in the boma control trial that differed from values at 5 min. The brackets indicate the time points at which the butorphanol + oxygen field trial differed from the control (a) or the butorphanol + oxygen boma trial differed from the control (c); (b) mean and s.d. of arterial blood pressure (mmHg) in field-immobilised rhinoceroses. The black arrow indicates the time at which butorphanol was administered and oxygen insufflation initiated. The asterisk (*) indicates values that differed from pre-intervention values (5 min), whilst the shaded area represents the normal range of the indirect mean arterial pressure in standing, unrestrained white rhinoceroses (Citino & Bush 2007).

Both field- and boma-immobilised rhinoceroses were tachycardic 5 min after initial recumbency (field-immobilised rhinoceroses 136 beats/min ± 34 beats/min, boma-immobilised rhinoceroses 141 beats/min ± 6 beats/min; normal range 32 beats/min – 42 beats/min, Figure 4a). The tachycardia was attenuated following administration of butorphanol + oxygen in field-immobilised rhinoceroses (heart rate 99 beats/min ± 30 beats/min at 10 min, *p* < 0.01, 5 min vs. 10 min). This decrease in heart rate was also observed in boma-immobilised rhinoceroses given the same treatment intervention (heart rate 92 beats/min ± 20 beats/min at 10 min,* p* < 0.01, 5 min vs. 10 min). Although heart rate continued to decrease in both boma- and field-immobilised rhinoceroses throughout the immobilisation period, they were still tachycardic (78 beats/min ± 20 beats/min and 70 beats/min ± 9 beats/min in field- and boma-immobilised rhinoceroses respectively) 20 min into the immobilisation period. There was no difference in heart rate between the boma treatment group (butorphanol + oxygen) and field-immobilised rhinoceroses. However, in both the field and boma trials rhinoceroses given butorphanol + oxygen had lower heart rates than those in the boma control trial at 10, 15 and 20 min [*F*_(2,28) _= 22.68, *p* < 0.0001], in spite of the decrease in heart rate over time in the control animals (139 beats/min ± 9 beats/min at 5 min versus 119 beats/min ± 18 beats/min at 20 min, *p* < 0.05).

### Arterial blood pressure

The field-immobilised rhinoceroses were initially hyper­tensive (mean arterial pressure [MAP] 158 mmHg ± 18 mmHg; normal range for indirect MAP 108 mmHg – 135 mmHg [Citino & Bush 2007], Figure 4b). Following the supportive intervention blood pressure immediately decreased to normotensive levels (MAP 114 mmHg ± 16 mmHg at 10 min, *p* < 0.01, 5 vs 10 min) and all values following butorphanol + oxygen were lower than pre-intervention [*F*_(3,42)_ = 42.8,* p* < 0.01]. There were no arterial blood pressure data from boma-immobilised animals for comparison.

## Ethical considerations

This project was approved by the Animal Use and Care Committee of South African National Parks (SANParks) as well as the Animal Ethics Screening Committee of the University of the Witwatersrand (clearance 2012/23/04). This trial was a continuation of a study in which the first phase was conducted in boma-held white rhinoceroses. The ARRIVE guidelines for reporting *in vivo* experiments were adhered to (Kilkenny *et al.* 2010).

## Discussion

Critical cardiorespiratory abnormalities that occur in chemically immobilised free-ranging white rhinoceroses can be improved significantly with the administration of intravenous butorphanol (a mixed opioid agonist-antagonist) at the beginning of the immobilisation, together with oxygen insufflation throughout the recumbent period. In a previous study of boma-immobilised rhinoceros it was shown that intravenous butorphanol with oxygen insufflation was better than butorphanol alone, oxygen insufflation alone, or a control in which no supportive treatment was administered, in reversing the hypoxaemia associated with opioid-induced immobilisation (Haw *et al.* 2014). Although the effects of immobilising and supportive drugs are probably best assessed in captive conditions where there is less stress and variability, results cannot be directly extrapolated to the field, and follow-up field studies are essential to assess the efficacy of treatments properly.

In the current study the effects of intravenous butorphanol with oxygen insufflation on cardiorespiratory responses of field-immobilised white rhinoceroses were evaluated for the first time and compared with the responses of boma-immobilised white rhinoceroses. The same protocol as that used in the boma study and a similar cohort of animals (sub-adult male white rhinoceroses) were used. The approach allowed investigation of whether a supportive intervention, demonstrated to improve hypoxaemia under tightly controlled conditions in a boma, was equally effective in field-immobilised animals. Compared with the trial in bomas, in the field darting drug doses (based on estimated animal mass) and body condition may vary more between individuals, whilst cardiorespiratory responses are also likely to be altered by increased exertional and psychological stress associated with helicopter darting.

It was found that butorphanol combined with oxygen insufflation indeed also improved oxygenation in field-immobilised white rhinoceroses. However, unlike boma-immobilised rhinoceroses, the hypoxaemia was not fully corrected. On the other hand, the chase before immobilisation in free-ranging rhinoceroses led to a less severe hypopnoea throughout immobilisation compared with the boma-immobilised rhinoceroses. The higher respiratory rate resulted in better ventilation, as demonstrated by the lower P_a_CO_2_ observed in field-immobilised compared with boma-immobilised rhinoceroses at 5 min. Ventilation further improved following the intervention. As respiratory rate increased only transiently after administration of butorphanol and oxygen, it is likely that an increased tidal volume together with decreased carbon dioxide production – parameters that were not measured in this study – accounted for the continued fall in P_a_CO_2 _over time.

Despite the lower partial pressure of carbon dioxide in arterial blood, field-immobilised rhinoceroses initially had a more severe acidaemia than their boma-immobilised counterparts, suggesting that metabolic acidosis was a significant contributor to the lower pH (DiBartola 2006). The low pH in the presence of low bicarbonate levels (< 22 mmol/L) also indicated that the acidosis was primarily of metabolic origin, as plasma bicarbonate moves into the cells to buffer the high levels of lactate (Kellum 2007). Although lactate concentrations were not measured in rhinoceroses in the boma trials, the high concentration of plasma lactate (11.4 mmol/L ± 5.2 mmol/L) in the field-immobilised rhinoceroses suggests that lactic acidosis was likely the primary contributor to the acidaemia. However, over time, and following administration of the supportive treatment, the pH increased in field-immobilised rhinoceroses such that by the end of the immobilisation period there was no difference in arterial pH between boma- and field-immobilised rhinoceroses. Plasma lactate concentration decreased from 11.4 ± 5.2 mmol/L at 10 min to 9.6 mmol/L ± 5.0 mmol/L at 20 min. It is difficult to discern whether the decreased lactate concentration reflects rapid metabolism of lactate over time or a response to the treatment intervention. P_a_CO_2_ in field-immobilised rhinoceroses also decreased from 63.0 mmol/L ± 7.7 mmHg at 5 min to 55.3 mmol/L ± 5.2 mmHg at 20 min. The improvement of pH was therefore probably a result of the combined effects of lactate metabolism, improved minute ventilation and decreased carbon dioxide production.

In parallel with respiratory depression and acid-base imbalances, immobilisation also resulted in altered cardiovascular function in free-ranging rhinoceroses. At the start of the immobilisation period (5 min), the rhinoceroses were severely tachycardic and moderately hypertensive. Administration of butorphanol and oxygen lowered the heart rate significantly, although normal values were not attained and heart rate remained about twice as high as that expected in standing, unsedated white rhinoceroses (Citino & Bush 2007). However, the initial hypertension completely resolved following administration of butorphanol and oxygen.

Although butorphanol and oxygen completely corrected parameters such as blood pressure, oxygenation was not entirely corrected in the field trial, according to suggested normal values obtained from unrestrained white rhinoceroses in similar environmental conditions (Citino & Bush 2007). However, the peak arterial oxygen concentration observed (81.2 mmol/L ± 23.7 mmHg) was higher than that found in other studies where partial reversal of etorphine and change in body position were used to improve oxygenation (Fahlman 2008; Miller *et al.* 2013; Wenger *et al.* 2007). Moreover, it has been suggested that rhinoceroses may be better able to maintain adequate tissue oxygenation with lower P_a_O_2_ values than smaller mammals can as a result of the rhinoceros's higher oxygen affinity for haemoglobin (lower P_50_) and lower tissue metabolic rate (Ball, Larsen & Wagman 2011; Baumann, Mazur & Braunitzer 1984; Heard *et al.* 1992). Therefore the improvement in arterial oxygenation in the rhinoceroses in this study may have been sufficient to prevent hypoxic damage to muscles and other vital organs, thereby reducing the risk of capture-related morbidity such as myopathy, renal failure, abortion and cardiac failure (Heard *et al.* 1992). Although lactate concentrations were high in field-immobilised rhinoceroses, it is believed that these elevated values were mainly as a result of the anaerobic metabolism during the physical exertion before immobilisation, rather than the lack of oxygen supply to cells during immobilisation, as lactate concentration decreased towards the end of the immobilisation period.

It is therefore believed that aerobic metabolism predominated by the end of the immobilisation period in both field- and boma-immobilised rhinoceroses. That P_a_O_2_ was completely corrected in the boma trial by the administration of butorphanol and oxygen, but not in the field trial, is somewhat surprising, but not entirely unexpected. Etorphine has been implicated in development of pulmonary hypertension (Heard *et al.* 1996; Meyer *et al.* 2015). In addition, activation of the sympathetic nervous system will induce pulmonary hypertension through vasoconstriction, via activation of alpha-1 and alpha-2 adrenoreceptors in the pulmonary vasculature (Hyman & Kadowitz 1986; Kadowitz & Hyman 1973; Salvi 1999). Similarly, hypoxia (Aaronson *et al.* 2006) and acidaemia (Harthoorn & Young 1976) also result in vasoconstriction in the pulmonary vasculature. Moreover, increased sympathetic tone will cause an increase in cardiac output resulting in greater blood flow to the lungs, thus further increasing pulmonary pressure in the vasoconstricted vessels.

It is hypothesised that in the field-immobilised rhinoceroses the psychological and physiological stress response associated with fleeing the helicopter resulted in a higher sympathetic outflow and pulmonary vasoconstriction compared with that in the habituated boma-immobilised rhinoceroses, resulting in more severe pulmonary hypertension. Pulmonary hypertension, leading to pulmonary congestion and possibly oedema (Gao & Raj 2005), will reduce oxygen diffusion from the alveoli into the arterial blood. Increased cardiac output resulting from sympathetic activation and exertion will also increase the speed of blood flow and result in decreased time for red blood cells to traverse alveolar capillaries in the pulmonary bed. If this transit time is less than 0.25 s, oxygen will not have enough time to diffuse and equilibrate in the blood (West 2012). Thus, functional changes in the pulmonary vasculature of field-immobilised white rhinoceroses may account for the differences observed in arterial oxygen concentration between field- and boma-immobilised rhinoceroses after they received identical supportive treatments.

In addition, the difference in arterial oxygen concentration between field- and boma-immobilised rhinoceroses may be attributed to differences in their metabolic rate at immobilisation. Animals with higher metabolism from exertion will have greater tissue oxygen consumption and lower venous oxygen contents, and hence greater partial pressure oxygen gradients between alveolar air and pulmonary arterial blood entering the pulmonary capillaries. In these field-immobilised rhinoceroses, with already compromised oxygen diffusion capabilities as described above, this increased gradient may have reduced the efficacy of the treatment intervention in oxygenating arterial blood. Although metabolism was not measured, it is known that anaerobic metabolism in field-immobilised rhinoceroses was significant, as evidenced by the high lactate values. In another study, where boma-held white rhinoceroses were immobilised with a combination of etorphine, azaperone and hyaluronidase, the highest median lactate value was 2.54 mmol/L (Buss *et al.* 2015), significantly lower than that found in the field-immobilised rhinoceroses (11.52 mmol/L ± 5.08 mmol/L).

Metabolic acidosis (characterised by low arterial pH, high lactate and low bicarbonate concentrations), together with a respiratory acidosis (characterised by low arterial pH and high P_a_CO_2_) contributed to the overall acidaemia observed in the field-immobilised rhinoceroses. However, metabolic acidosis was most likely the primary contributor to the acidosis as the hypercapnia was relatively mild. In the field-immobilised rhinoceroses both the hypercapnia and acidaemia improved from 5 min (pre-intervention) to 10 min (4 min after administration of butorphanol and oxygen). Surprisingly, in a previous study where oxygen insufflation was used on its own to improve arterial oxygenation in field-immobilised white rhinoceroses, the initial acidaemia and hypercapnia did not improve (Bush *et al.* 2004). Thus, butorphanol combined with oxygen appears superior to oxygen insufflation alone in field-immobilised white rhinoceroses.

Results from another recent study (Miller *et al.* 2013) indicated that butorphanol may be better administered in the dart mixture rather than after immobilisation has occurred. The authors showed that when butorphanol was administered to the rhinoceros in the immobilising dart mixture, the rhinoceros's plasma lactate concentration was lower and acidaemia less severe compared with when it was administered subsequent to immobilisation. The authors concluded that butorphanol in the dart mixture led to a decreased intensity of exertion during induction, which resulted in less severe metabolic derangements. The finding that plasma lactate concentration was positively correlated with the speed at which the rhinoceros ran after dart placement supports the view that decreased intensity of exertion during induction will decrease the severity of the acidaemia. However, rhinoceroses were still severely hypoxaemic in the study when butorphanol was administered with the dart mixture (Miller *et al.* 2013). Further studies are necessary to determine whether butorphanol administered in the dart, together with oxygen administered during recumbency, would result in improved acid-base balance together with increased oxygenation in immobilised white rhinoceroses.

In addition to arterial blood gas abnormalities, etorphine-induced immobilisation in white rhinoceroses has been known to induce hypertension, as observed in this study (Heard *et al.* 1992; Kock *et al.* 1995; LeBlanc *et al.* 1987). Although no definitive mechanism has been identified, tachycardia, peripheral vasoconstriction, or increased sympathetic tone (as a result of hypoxia, exercise or the drug effect) are suggested factors implicated in etorphine-induced hypertension in both rhinoceroses and equids (Daniel & Ling 1972; Heard *et al.* 1992). Azaperone is therefore routinely used in rhinoceros immobilisation combinations for its sedative effects as well as its alpha-1 antagonistic effect, which blocks vasoconstriction induced by opioids (Burroughs *et al.* 2012).

That the tachycardia and hypertension improved in parallel with the improvement in arterial oxygenation following treatment with butorphanol and oxygen suggests that the hypoxia-induced sympathetic response was a predominant factor implicated in the initial hypertension and tachycardia (Schultz, Li & Ding 2007). However, a direct drug effect may also account for the observed changes. Intravenous administration of butorphanol in game-ranched etorphine-immobilised white rhinoceroses has been shown to decrease heart rate and systemic blood pressure (Boardman *et al.* 2014). Similarly, in free-ranging white rhinoceroses, intravenous butorphanol resulted in a decrease in heart rate in etorphine-immobilised white rhinoceros (Miller *et al.* 2013).

Surprisingly, in a study where oxygen insufflation was used to correct etorphine-induced hypoxaemia in the immobilised white rhinoceros, heart rate and blood pressure did not improve with administration of oxygen, despite the increase in arterial oxygenation (Bush *et al.* 2004). It is therefore suggested that butorphanol's significant activity at opioid receptors (Commisky *et al.* 2005) may have a greater effect on the cardiovascular system than the improvement in arterial oxygen concentration alone. It cannot, however, be discounted that some of the observed cardiovascular effects may have been attributed to the azaperone in the dart mixture, which has a longer onset of action than etorphine (Portas 2004), with a peak effect occurring one hour after administration (ZooPharm 2013).

Mortalities of white rhinoceros under etorphine immobilisation have been reported, with worsening hypoxaemia noted as a risk factor for mortality (Kock *et al.* 1995). Therefore the protocol of administering intravenous butorphanol together with oxygen via nasotracheal intubation, which significantly improved oxygenation and reduced hypercapnia, acidaemia and heart rate, would undoubtedly reduce the risk of immobilisation-related morbidity and mortality in field-immobilised white rhinoceroses. To further reduce this risk it is believed that refinement of butorphanol doses, oxygen flow rates and administration methods should be considered.

In addition, further research is needed to clarify which physiological disturbances cause worse respiratory compromise and hypoxia in chased compared to non-chased animals after treatment. Once these pathophysiological effects have been clarified, alternative treatment options aimed at correcting the specific physiological imbalances can be implemented.

## Conclusion

The efficacy of oxygen insufflation combined with intravenous butorphanol, administered at the beginning of the immobilisation period, in treating cardiorespiratory compromise in field-immobilised white rhinoceroses was evaluated. This treatment protocol was based on the findings of a previous boma study, where butorphanol and oxygen completely corrected the immobilisation-induced hypoxaemia.

Despite the additional physiological imbalances that occur during field-immobilisations, this treatment intervention was still superior to previous protocols aimed at correcting immobilisation-induced hypoxaemia. Administering oxygen with intravenous butorphanol is a simple, readily available technique that will significantly improve oxygenation, thereby improving the safety of white rhinoceros immobilisation.
